# Extracellular RNA communication: A decade of NIH common fund support illuminates exRNA biology

**DOI:** 10.1002/jev2.70016

**Published:** 2025-01-16

**Authors:** Sara M. Amolegbe, Nicolas C. Johnston, Angela Ambrosi, Aniruddha Ganguly, T. Kevin Howcroft, Lillian S. Kuo, Patricia A. Labosky, Dobrila D. Rudnicki, John S. Satterlee, Danilo A. Tagle, Christine Happel

**Affiliations:** ^1^ Office of the Director National Institutes of Health Bethesda Maryland USA; ^2^ National Institute on Drug Abuse National Institutes of Health Bethesda Maryland USA; ^3^ National Cancer Institute National Institutes of Health Bethesda Maryland USA; ^4^ National Center for Advancing Translational Sciences National Institutes of Health Bethesda Maryland USA

**Keywords:** exosome, exRNA, extracellular RNA, extracellular vesicle, NIH

## Abstract

The discovery that extracellular RNAs (exRNA) can act as endocrine signalling molecules established a novel paradigm in intercellular communication. ExRNAs can be transported, both locally and systemically in virtually all body fluids. In association with an array of carrier vehicles of varying complexity, exRNA can alter target cell phenotype. This highlights the important role secreted exRNAs have in regulating human health and disease. The NIH Common Fund exRNA Communication program was established in 2012 to accelerate and catalyze progress in the exRNA biology field. The program addressed both exRNA and exRNA carriers, and served to generate foundational knowledge for the field from basic exRNA biology to future potential clinical applications as biomarkers and therapeutics. To address scientific challenges, the exRNA Communication program developed novel tools and technologies to isolate exRNA carriers and analyze their cargo. Here, we discuss the outcomes of the NIH Common Fund exRNA Communication program, as well as the evolution of exRNA as a scientific field through the analysis of scientific publications and NIH funding. ExRNA and associated carriers have potential clinical use as biomarkers, diagnostics, and therapeutics. Recent translational applications include exRNA‐related technologies repurposed as novel diagnostics in response to the COVID‐19 pandemic, the clinical use of extracellular vesicle‐based biomarker assays, and exRNA carriers as drug delivery platforms. This comprehensive landscape analysis illustrates how discoveries and innovations in exRNA biology are being translated both into the commercial market and the clinic. Analysis of program outcomes and NIH funding trends demonstrate the impact of this NIH Common Fund program.

## Graphical abstract

The NIH Common Fund exRNA Communication program was established in 2012 to accelerate and catalyze progress in the exRNA biology field. The program addressed both exRNA and exRNA carriers, and served to generate foundational knowledge for the field from basic exRNA biology to future potential clinical applications. The Extracellular RNA Communication program goals for Phase I (blue), Phase II (red), and combined Phase I/II (purple) are indicated in the graphical abstract. Here we discuss outcomes of the NIH Common Fund exRNA Communication program, as well as the evolution of exRNA as a scientific field.

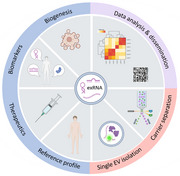

## 1 THE EXTRACELLULAR RNA COMMUNICATION PROGRAM

RNA is an essential molecule that is fundamental for life. It carries messages from the nucleus to the cell's molecular machinery for translation into proteins. In addition to its coding role, RNA can have non‐coding functions that include enzymatic activity and regulation of gene expression at the transcriptional, post‐transcriptional levels, and post‐translational levels. Extracellular RNA (exRNA) refers to RNA found outside of the cells in which it was transcribed. Most exRNAs are transported and protected from degradation in the extracellular spaces through encapsulation in extracellular vesicles or through interaction with a variety of other nanoparticles, and in some cases may carry messages from one cell to another. ExRNA was first detected in human blood in 1944 (Mandel & Metais, 1948), but it was initially unclear what role, if any, exRNA and its carriers played in biology. However, several compelling discoveries at the beginning of the millennium increased scientific interest and raised awareness of exRNA as a developing area of biology (Alvarez‐Erviti et al., 2011; Howcroft et al., 2011; Waldenström et al., 2012; Zhang et al., 2012; Zhuang et al., 2011). Each new discovery brought new and perplexing questions, and the field of ExRNA biology struggled to reach consensus. For example, researchers wanted to learn more about how exRNAs are involved in diseases like cancer, heart disease, or neurological diseases like Alzheimer's, and how exRNAs can be used to diagnose or even treat diseases. The NIH Common Fund's Extracellular RNA Communication program aimed to unlock the mysteries of exRNAs, including how exRNAs are made and function, and how they communicate within the body to uncover their roles in human health and disease.

In 2013, the National Institutes of Health (NIH) launched the Extracellular RNA Communication Common Fund program. The NIH Common Fund is a funding entity within NIH managed by the Office of Strategic Coordination in the Division of Program Coordination, Planning and Strategic Coordination in the Office of the NIH Director. Common Fund programs explore emerging scientific questions and opportunities and address important, high‐priority problems in biomedical research that no single NIH Institute or Center can address on its own. This unique resource supports innovative, high‐risk and potentially high‐impact research through short‐term, goal‐driven programs intended to change scientific paradigms and catalyze research across multiple disciplines.

**FIGURE 1:**
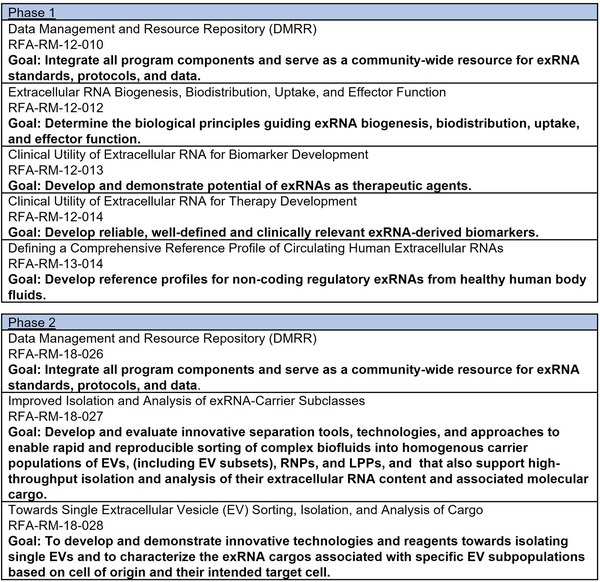
Extracellular RNA Communication program goals. The funding opportunities and programmatic goals for Phases 1 and 2 of the exRNA Communication program are summarized. Extracellular vesicles, ribonucleoproteins (RNPs) and lipoproteins (LPPs).

The Extracellular RNA Communication program was launched to accelerate progress in the field of exRNA biology. Programmatic goals for Phase 1 of the Extracellular RNA Communication program aimed to establish fundamental biological principles of extracellular RNA secretion, delivery and impact on recipient cells (Figure 1). The program also aimed to catalogue exRNAs in human biofluids and to explore the extent to which exRNAs from non‐human cells were present in these biofluids. Understanding exRNAs in human biofluids informed the goal of testing the clinical utility of exRNAs to diagnose, monitor and treat disease. Finally, the program was established to provide an accessible data and resource repository to the broader research community. Researchers funded through this program worked together as the Extracellular Communication Consortium (ERCC), made up of researchers from a diverse array of institutions across the United States, and collaborations were initiated across disciplines to explore this developing field (Ainsztein et al., 2015).

Phase 2 of the program was launched in 2019 and built on the successes of Phase 1 (Mateescu et al., 2022). One of the most daunting obstacles to the field was the inability to precisely detect and measure exRNAs in biofluids (Li et al., 2018). The inability to quickly isolate extracellular vesicles from biofluids limited their utility in diagnosing and treating disease. Consequently, Phase 2 was primarily focused on technology development, aiming to improve separation technologies and single extracellular vesicle analysis tools. Projects funded during Phase 2 aimed to rapidly sort carrier subclasses and identify their respective exRNA cargo (or exRNAomes); enable mapping exRNAs to their cell of origin; assess exRNA extracellular vesicle heterogeneity and further build and maintain tools and resources developed through the program for use by the scientific community (Figure 1).

Since its founding, the Extracellular RNA Communication program has established a remarkable record of achievements. The ERCC established data standards, a data portal and developed tools and reagents, many of which are available to the scientific community and described in detail below. ERCC researchers catalogued exRNA molecules found in human biofluids—including blood, plasma, saliva and urine—from over 2000 donors (Freedman et al., 2016; Godoy et al., 2018). They also identified potential exRNA biomarkers for nearly 30 diseases and conditions, including neurological disorders, cardiovascular disease, pregnancy complications, glaucoma, diabetes and multiple types of cancer (Danielson et al., 2018; Ghai et al., 2019; Li et al., 2018; Lucero et al., 2020; Srinivasan et al., 2020). ERCC researchers have also made substantial progress towards creating better separation technologies and tools for single extracellular vesicle analysis (Andronico et al., 2021; Gu et al., 2021; Lennon et al., 2022; Murillo et al., 2019; Oliveira et al., 2020; Wang et al., 2021; Welsh et al., 2022).

A total of 30 projects were funded in Phase 1 of the program, and 12 projects were funded in Phase 2. This includes a Data Management and Resource Repository (DMRR) which launched at the beginning of Phase 1 and continued through Phase 2. A summary of the notice of funding opportunities (NOFOs) and goals can be found in Figure 1. All awards were made through cooperative agreements, which requires substantial NIH scientific and programmatic involvement with the awardees. Under a cooperative agreement, NIH staff support and stimulate the recipient's milestone‐driven research but do not assume direction or prime responsibility for the research.

The DMRR developed the exRNA Portal, available at exrna.org. This unique resource provides information about the exRNA Communication program and links researchers to useful tools, educational materials and repositories. The portal includes over thirty detailed protocols for biofluid collection, extracellular vesicle isolation and enrichment, RNA sequencing and isolation, validation and quantification and extracellular vesicle proteomics. Additionally, the ERCC developed resources to help researchers select the best tools for their approach, including protocol selection guides, a technology selection matrix, and an extracellular vesicle antibody database (Rozowsky et al., 2019; Srinivasan et al., 2019). The portal also links to educational resources such as courses and seminars, as well as data repositories such as the NanoFlow repository and the exRNA Atlas. See Table 1 for information and links to these and other selected resources developed by the ERCC.

**TABLE 1:** Select list of resources developed with support from the NIH Common Fund Extracellular RNA Communication program. List of resources created by ERCC investigators and the exRNA Data Management and Resource Repository (DMRR).

Resource​	Brief description​	Link​
exRNA Research Portal​	The exRNA Research Portal is the information hub of the exRNA Communication program. ​	https://exrna.org/​
exRNA Atlas​	ERCC data repository that includes small RNA sequencing and qPCR‐derived exRNA profiles from human and mouse biofluids.​	https://exrna‐atlas.org/​
miRDaR​	miRDaR is a web application that enables customized selection of optimal exRNA isolation methods.​	https://exrna.shinyapps.io/mirdar/​
exceRpt​	ExeRpt is a structured pipeline for analyzing exRNA‐seq datasets and the exRNA‐processing toolkit of the ERCC.​	https://genboree.org/theCommons/projects/exr na‐tools‐may2014​
Extracellular vesicle Antibody database​	Antibody database designed to collect and share information on curated antibodies and protocols for targets relevant to extracellular vesicle biology.​	https://exrna.org/resources/evabdb/​
Nanoflow repository​	Data repository that provides a bioinformatics infrastructure for nanoflow data and informatics tools for nanoflow cytometry.​	https://genboree.org/nano‐ui/​
Technology matrix​	Interactive guide comparing a variety of technologies for the study of exRNA.​	https://exrna.org/resources/ercc2‐tech‐query/​
exRNA protocols​	Collection of protocols and standard operating procedures developed and refined by the ERCC.​	https://exrna.org/resources/protocols/​
exRNA outreach​	Collection of ERCC‐developed exRNA courses, ERCC webinar series, workshops and seminar recordings.​	https://exrna.org/resources/education/​

### 1.1 A decade of exRNA funding

**FIGURE 2:**
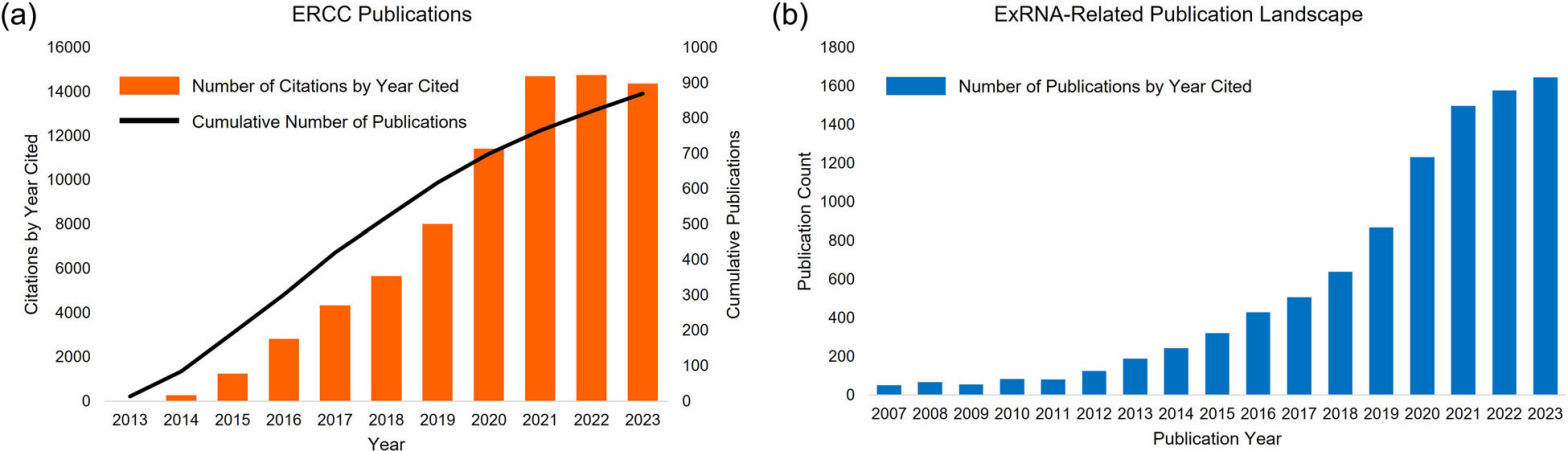
ExRNA publications. (a) Cumulative count of ERCC publications and citations by the year they were cited. The search was performed on 18 March 2024, using NIH's iCite tool by searching all publications associated with ExRNA Common Fund program grant numbers. (b) Number of exRNA‐related publications published between 2007 and 2023. The search was performed on 18 March 2024 using NIH's iSearch tool using the following search terms: ‘exRNA’ OR ‘extracellular RNA’ OR (‘extracellular vesicle’ AND ‘RNA’) OR ‘RNA communication’ OR ‘secreted RNA’ OR ‘circulating RNA’ OR (‘Exosome’ AND ‘RNA’). Search fields: Title, Abstract, PubMed Keywords.

ExRNA Communication program awardees have published prolifically, with manuscripts in over 100 different scientific journals, demonstrating the broad reach of the program into various biomedical specialties. As of March 2024, based on acknowledgement of NIH ExRNA Common Fund grant numbers, the ExRNA program has published 877 papers which were cited more than 79,442 times in peer‐reviewed literature (Figure 2a).

Using the mean relative citation ratio (RCR) (Hutchins et al., 2016), a metric developed by the NIH Office of Portfolio Analysis as a benchmark to assess influence relative to other papers using a field‐ and time‐normalized citation rate, the ERCC publication set has a mean RCR of 4.87. This suggests that publications from the program were cited on average 4.87 times more than the median NIH‐funded publication, significantly exceeding the benchmark measure of scientific influence for similar papers. The top cited exRNA‐related research articles from the ERCC are listed in Table 2. These original articles all have an RCR of greater than 10, up to 88.09, indicating that these papers are highly influential in the field of exRNA communication research. The ExRNA program publications cover a broad range of topics, addressing many goals of the Common Fund exRNA Communication program. Table 3 provides a selected subset of papers illustrating progress towards the overall program goals (Figure 1).

**TABLE 2:** Top cited exRNA‐related research articles from the ERCC. The search was performed using NIH's iSearch tool using grant numbers associated with the ExRNA Common Fund program. Reviews and position papers were excluded. Original articles were then manually reviewed to include only articles that primarily involved research relating to exRNA. Search date: 17 January 2024. Relative citation ratio (RCR) and total citations are listed for each publication.

Year	Title	Journal	Reference	RCR	Total citations
2019	Reassessment of Exosome Composition.	*Cell*	Jeppesen et al. (2019)	88.09	1463
2014	Cancer exosomes perform cell‐independent microRNA biogenesis and promote tumorigenesis.	*Cancer Cell*	Melo et al. (2014)	37.08	1106
2019	Suppression of Exosomal PD‐L1 Induces Systemic Anti‐tumour Immunity and Memory.	*Cell*	Poggio et al. (2019)	35.78	709
2014	Dynamic biodistribution of extracellular vesicles in vivo using a multimodal imaging reporter.	*ACS Nano*	Lai et al. (2014)	19.27	556
2015	The landscape of microRNA, Piwi‐interacting RNA, and circular RNA in human saliva.	*Clin Chem*	Bahn et al. (2015)	18.08	491
2016	High‐resolution proteomic and lipidomic analysis of exosomes and microvesicles from different cell sources.	*J Extracell Vesicles*	Haraszti et al. (2016)	17.97	418
2015	Statistically based splicing detection reveals neural enrichment and tissue‐specific induction of circular RNA during human fetal development.	*Genome Biol*	Szabo et al. (2015)	13.79	409
2015	Directional cell movement through tissues is controlled by exosome secretion.	*Nat Commun*	Sung et al. (2015)	13.26	389
2018	Plant‐Derived Exosomal MicroRNAs Shape the Gut Microbiota.	*Cell Host Microbe*	Teng et al. (2018)	19.67	383
2015	Chip‐based analysis of exosomal mRNA mediating drug resistance in glioblastoma.	*Nat Commun*	Shao et al. (2015)	14.17	380

**TABLE 3:** A selected subset of papers that demonstrate progress towards the goals of the exRNA Communication program. Select ERCC‐funded publications are highlighted in three main areas that align with the goals of the exRNA Communication program.

**exRNA data and resources**	**PMID**	**Reference**
Murillo *et al*. exRNA Atlas Analysis Reveals Distinct Extracellular RNA Cargo Types and Their Carriers Present across Human Biofluids. Cell. 2019 Apr 4;177(2):463‐477.e15.	30951672	Murillo et al. (2019)
Rozowsky *et al*. exceRpt: A Comprehensive Analytic Platform for Extracellular RNA Profiling. Cell Syst. 2019 Apr 24;8(4):352‐357.e3.	30951672	Rozowsky et al. (2019)
LaPlante *et al*. exRNA‐eCLIP intersection analysis reveals a map of extracellular RNA binding proteins and associated RNAs across major human biofluids and carriers. Cell Genom. 2023 Apr 20;3(5):100303.	37228754	LaPlante et al. (2023)
**Advances in ExRNA Biology**		
Freedman *et al*. Diverse human extracellular RNAs are widely detected in human plasma. Nat Commun. 2016 Apr 26;7:11106.	27112789	Freedman et al. (2016)
Godoy *et al*. Large Differences in Small RNA Composition Between Human Biofluids. Cell Rep. 2018 Oct 30;25(5):1346‐1358.	30380423	Godoy et al. (2018)
Zhang *et al*. Supermeres are functional extracellular nanoparticles replete with disease biomarkers and therapeutic targets. Nat Cell Biol. 2021 Dec;23(12):1240‐1254.	34887515	Zhang et al. (2021)
**Enabling technologies **		
Andronico *et al*. Sizing Extracellular Vesicles Using Membrane Dyes and a Single Molecule‐Sensitive Flow Analyzer. Anal Chem. 2021 Apr 13;93(14):5897‐5905.	33784071	Andronico et al. (2021)
Gu *et al*. Acoustofluidic centrifuge for nanoparticle enrichment and separation. Sci Adv. 2021 Jan 1;7(1):eabc0467.	33523836	Gu et al. (2021)
Wang *et al*. Slowing down DNA translocation through solid‐state nanopores by edge‐field leakage. Nat Commun. 2021 Jan 8;12(1):140.	33420061	Wang et al. (2021)

ExRNA‐related publication numbers, from within and outside the Common Fund program, have increased steadily since the Common Fund ExRNA program made its first awards in 2013 (Figure 2b). Although an increase in publications from the broader scientific community is not a direct result of the Common Fund program, this illustrates that the field of extracellular vesicles and exRNA was still considered an emerging area of research when the NIH Common Fund ExRNA program launched in 2013 and is consistent with the idea that the investment helped accelerate a rapidly growing field of important biomedical research.

**FIGURE 3:**
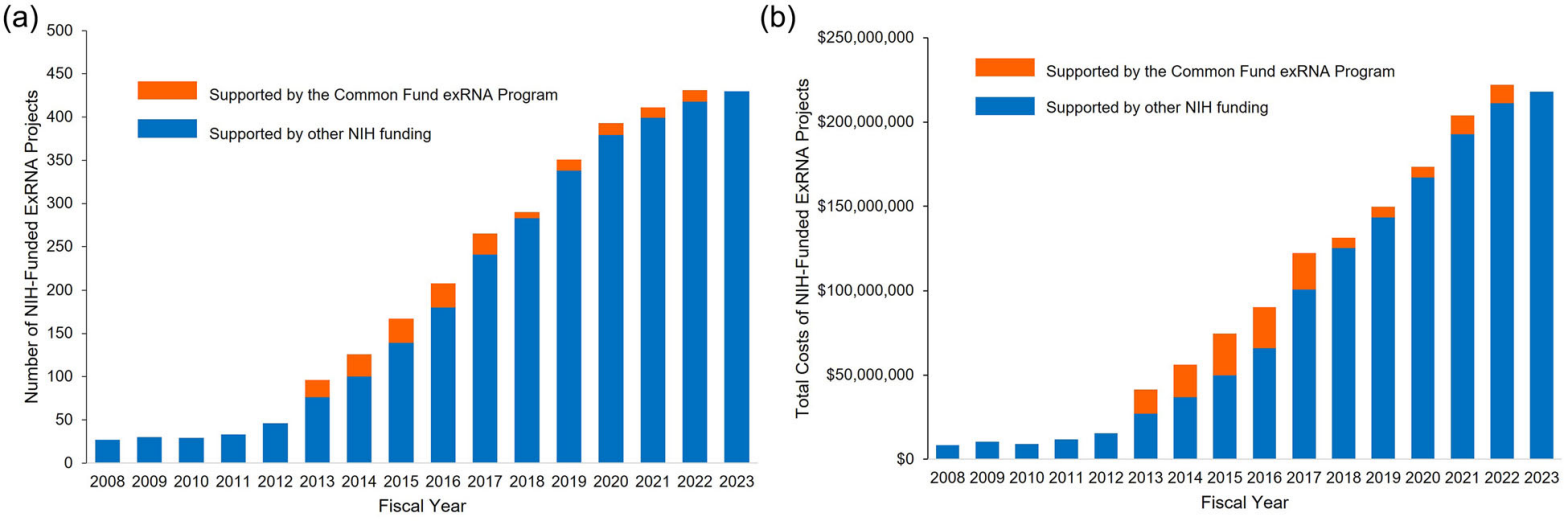
NIH funded exRNA‐related projects. (a) Number of exRNA‐related NIH awards funded between 2008 and 2023. (b) Total costs awarded to exRNA‐related project for all NIH awards between 2008 and 2023. Both searches were performed using NIH's internal iSearch tool using the following search terms: ‘exRNA’ OR ‘extracellular RNA’ OR (‘extracellular vesicle’ AND ‘RNA’) OR ‘RNA communication’ OR ‘secreted RNA’ OR ‘circulating RNA’ OR (‘Exosome’ AND ‘RNA’). Search Fields: Title; Abstract; Specific Aims. Both figures display competing and non‐competing awards. In (a), this means that the number of projects by year is displayed, so a multiyear grant would be displayed each year it received additional funding. Administrative supplements to existing grants were excluded from the project count but included in total costs. Search date: 14 March 2024.

A portfolio analysis of NIH research grants in the exRNA research area was performed to quantify the NIH investment in this area, both inside and outside the Common Fund program. This analysis revealed that investment in exRNA research has increased in NIH Institutes and Centers (ICs) independent of Common Fund support since the program began. Prior to the start of the Common Fund program in 2013, NIH investment in this field was approximately $55 million between 2008 and 2012. Since 2013, the total number of exRNA‐related NIH awards and the total costs awarded to exRNA‐related projects have gone up each year, with over $515 million invested between 2013 and 2018, and more than $965 million invested between 2019 and 2023 (Figure 3).

**FIGURE 4:**
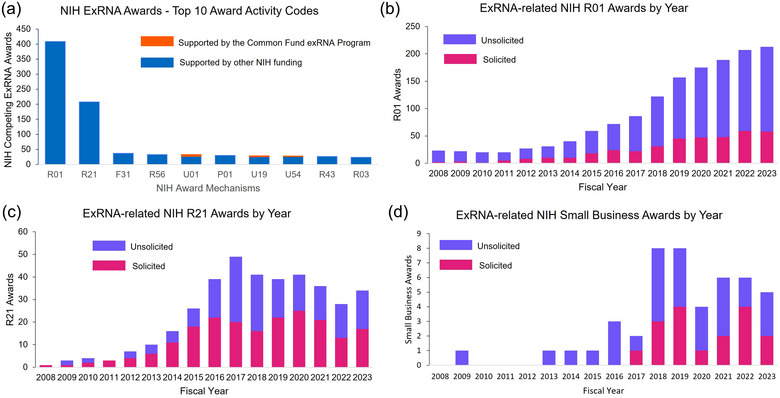
Types of NIH funded exRNA‐related awards. (a) All exRNA‐related NIH awards by activity code. Data in (a) includes only new (competing) awards. (b–d) NIH solicited and unsolicited R01 (b), R21 (c) and R41/R42/R43/R44 (d) awards (SBIR/STTR) related to extracellular RNA communication made each fiscal year from 2008 to 2023. Searches were performed using NIH's iSearch tool using the following terms: ‘exRNA’ OR ‘extracellular RNA’ OR (‘extracellular vesicle’ AND ‘RNA’) OR ‘RNA communication’ OR ‘secreted RNA’ OR ‘circulating RNA’ OR (‘Exosome’ AND ‘RNA’). Search fields: Title; Abstract; Specific Aims. (b–d) display competing and non‐competing awards to show number of projects by year. Administrative supplements to existing awards were excluded from the project count. Search date: 14 March 2024.

While the ExRNA Common Fund program mainly utilized the cooperative agreement funding mechanism, NIH overall has primarily awarded exRNA‐related projects using the R01 (Research Project) and R21 (Exploratory/Developmental Research) grant mechanisms, two of the most common NIH award types (Figure 4a) (National Institutes of Health, 2024). Typically, successful R01 applications have robust preliminary data supporting a hypothesis; R01 awards are usually 5 years in duration with an average total cost of $609,790 (National Institutes of Health, 2023). R21 applications are typically for exploratory studies that may break new ground and are often considered higher risk than traditional R01 proposals; they are usually awarded for 2 years and cannot exceed $275,000 in total costs. Examining exRNA‐related R01 and R21 awards over time, both for solicited (NIH requests grants in a given research area) and unsolicited (investigator‐initiated) awards, shows an increase in R21 awards starting around 2012 to a maximum in 2017 (Figure 4c). The number of exRNA related R01 awards also increased over time, particularly after 2017 (Figure 4b). These data are consistent with the possibility that exploratory research done through R21s could have progressed into related R01s in the years following. A greater number of unsolicited R01 awards were made each year compared to solicited awards, with an overall increasing trend over time, which suggests that more investigators are independently proposing research exploring exRNA communication and those awards are funded at a higher rate than awards made based on NIH requests for applications in this area. Although fewer than ten exRNA‐related NIH small business awards were made before 2017, we have seen a marked increase these types of awards since then (Figure 4d).

**FIGURE 5:**
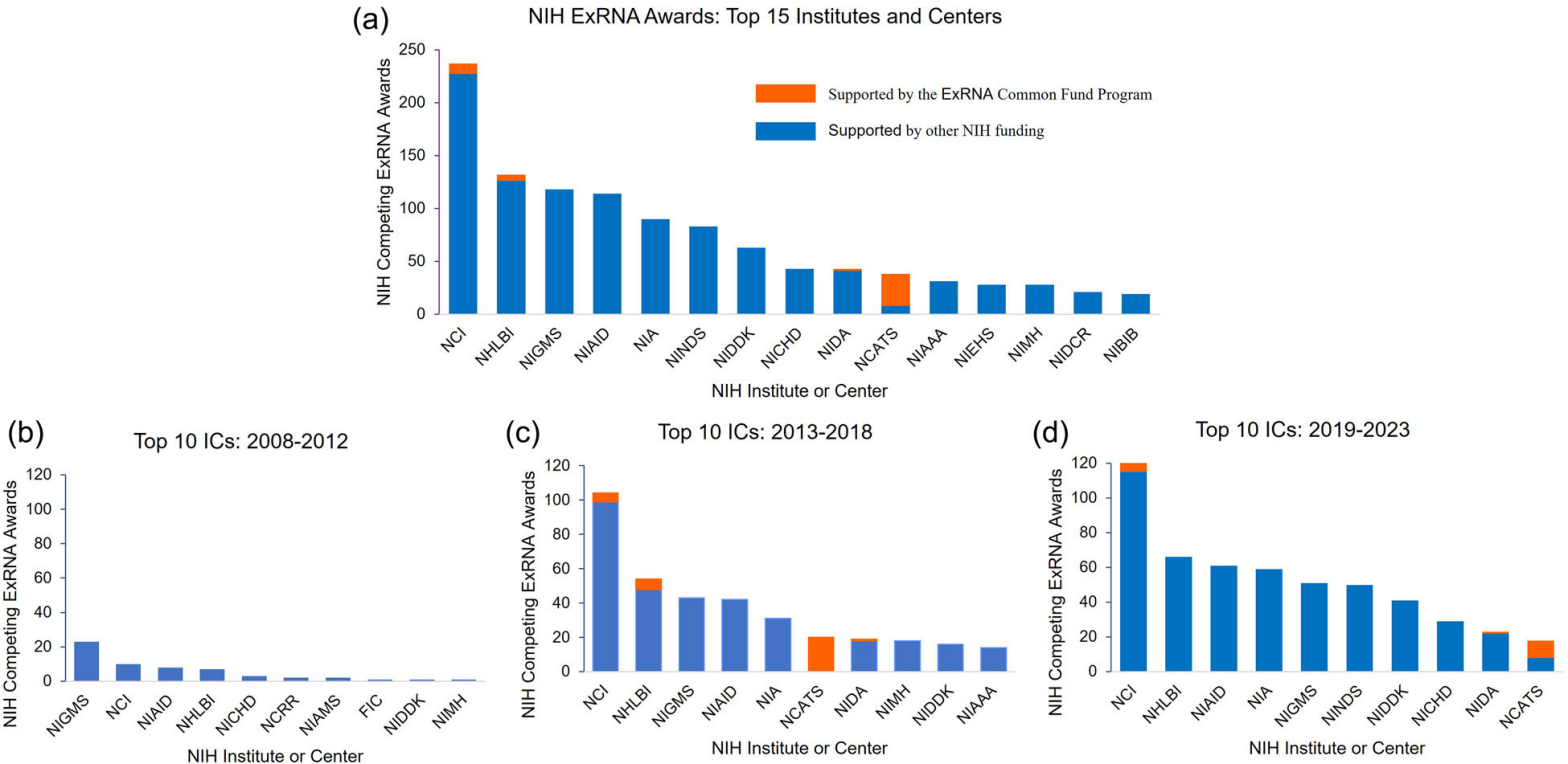
ExRNA‐related awards across administering NIH Institutes and Centers. (a) overall; (b) from 2008 to 2012 before the Common Fund ExRNA program; (c) from 2013 to 2018 during Phase I of the Common Fund ExRNA program and (d) 2019–2023 during Phase II of the Common Fund ExRNA program. (a–d) Searches were performed using NIH's internal iSearch tool using the following terms: ‘exRNA’ OR ‘extracellular RNA’ OR (‘extracellular vesicle’ AND ‘RNA’) OR ‘RNA communication’ OR ‘secreted RNA’ OR ‘circulating RNA’ OR (‘Exosome’ AND ‘RNA’). Search Fields: Title; Abstract; Specific Aims. Figures display only new (competing) awards (Types 1 and 2 NIH grants). Search date: 14 March 2024.

The total number of exRNA‐related awards has also increased over time across several NIH ICs illustrating the growth of this area of science. Figure 5 compares the numbers of awards across NIH Institutes and Centers over time. From 2018 to 2022, the National Cancer Institute (NCI); National Institute on Aging (NIA) and National Heart, Lung, and Blood Institute (NHLBI) made the most exRNA related awards. NCI had 10 exRNA‐related projects between 2008 and 2012, but that increased to 120 between 2019 and 2023. Over the same time, exRNA‐related projects from NIA increased from 0 to 59 projects, and from NHLBI increased from 7 to 66 projects. The broad increase across many ICs suggests increasing interest and acceptance of extracellular RNA related projects in many different fields under the NIH umbrella.

### 1.2 Peer review

Peer review is a cornerstone of the NIH extramural research mission. It ensures that all applications submitted to NIH are evaluated by scientific experts in a fair and unbiased manner. The first level of review is carried out by a Scientific Review Group (SRG; also referred to as study section). Study sections are primarily composed of non‐federal scientists with expertise in relevant scientific disciplines. The second level of review is performed by NIH Institute and Center National Advisory Councils or Boards. NIH applications are assigned to specific study sections based on the expertise needed to evaluate the scientific and technical merit of the application. Chartered study sections review the majority of investigator‐initiated research applications. Special Emphasis Panels (SEPs) are ad hoc NIH study sections held to review applications on special topics and member conflict applications. SEPs can be standalone or recurring meetings, and recruit reviewers based on the expertise needed at each meeting. As such, SEPs include only temporary members (National Institutes of Health Center for Scientific Review, 2024).

**FIGURE 6:**
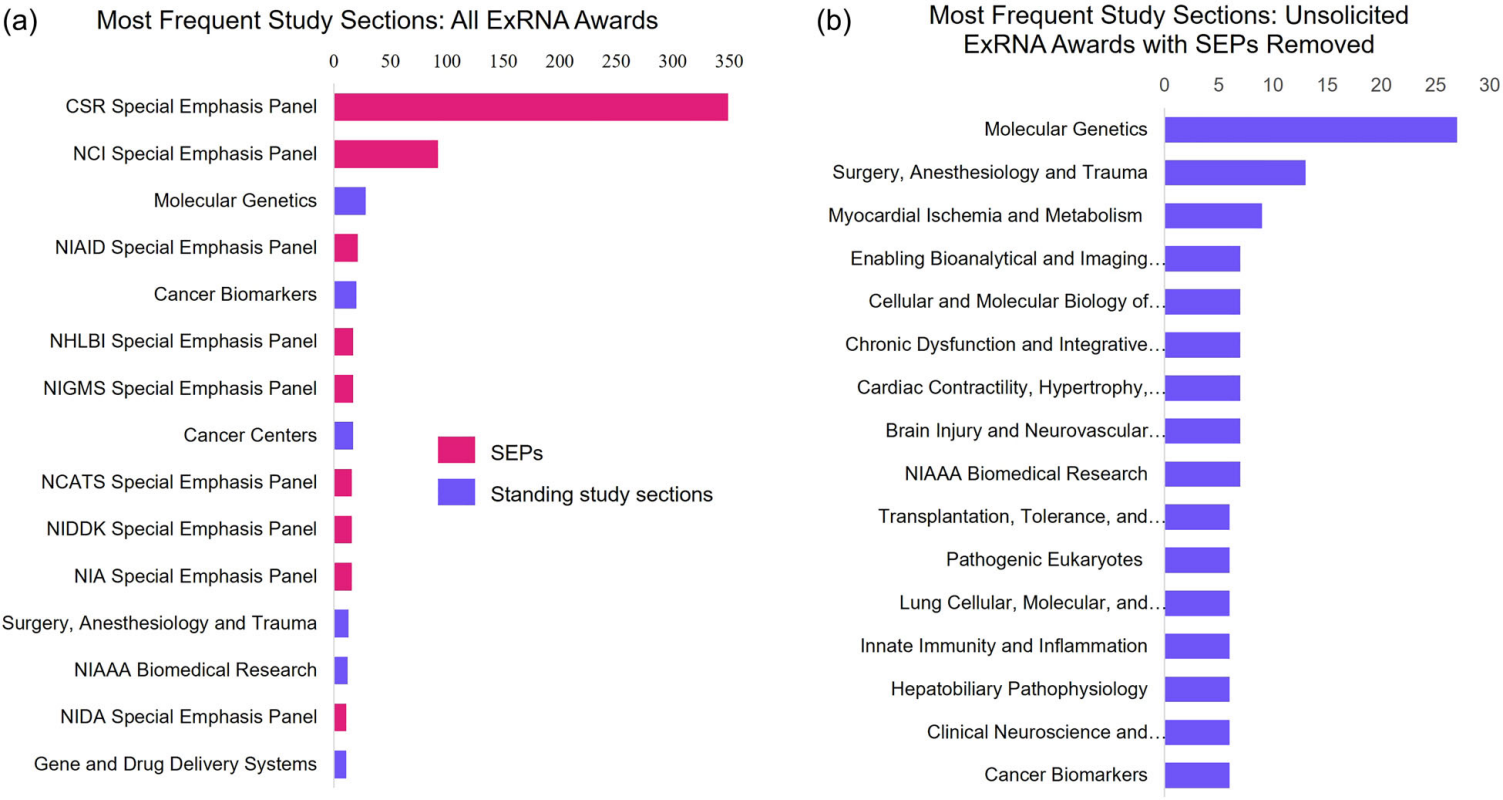
Most frequent NIH study sections for exRNA‐related awards. (a) Most frequent study sections where competing exRNA‐related NIH awards were reviewed from 2008 to the search date (14 March 2024). (b) Most frequent study sections where competing unsolicited exRNA‐related NIH awards were reviewed after removing Special Emphasis Panels. Searches were performed using NIH's internal iSearch tool using the following terms: ‘exRNA’ OR ‘extracellular RNA’ OR (‘extracellular vesicle’ AND ‘RNA’) OR ‘RNA communication’ OR ‘secreted RNA’ OR ‘circulating RNA’ OR (‘Exosome’ AND ‘RNA’). Search Fields: Title; Abstract; Specific Aims. Figures display only competing awards (Types 1 and 2 NIH grants). Search date: 14 March 2024.

In Figure 6a, we show the most frequent study sections where competing exRNA‐related NIH awards were reviewed. We found that exRNA‐related awards were overwhelmingly reviewed in SEPs. SEPs represented 9 out of the 15 most frequent study sections. The NIH Center for Scientific Review (CSR) SEP has reviewed the largest number of awarded applications. This suggests that many exRNA‐related awards were associated with special topic areas or areas of emphasis that necessitated the establishment of an SEP. NIH SEPs are often convened in response to IC‐sponsored funding opportunities focused on areas of increased priority and/or emphasis for a specific area of science. In Figure 6b, only unsolicited awards are shown and SEPs are removed, providing information on the standing study sections that most often reviewed exRNA‐related awarded applications. While the Molecular Genetics study section reviewed the most applications that were ultimately awarded, exRNA‐related awards were overall reviewed in study sections focusing on a broad and diverse range of scientific disciplines. This demonstrates that exRNA research applies broadly to multiple areas of the biomedical research enterprise with a number of diverse applications.

## 2 Translating exRNA and extracellular vesicle knowledge into commercial products

Knowledge generated by the ERCC on the biology of exRNA and extracellular vesicles, along with technological advances, has provided a platform for the development of novel exRNA and extracellular vesicle‐based tools and applications. ExRNAs and extracellular vesicles can be harnessed for clinical use as biomarkers, diagnostics and therapeutics. The stability and relative availability of exRNA molecules makes them an interesting class of biomarkers. Real time changes in exRNA expression offer prognostic values in predicting disease outcomes, monitoring treatment response and assessing treatment risk (Hornick et al., 2015; Marar et al., 2021; Mehta et al., 2010). Extracellular vesicles can also serve as natural carriers for therapeutic agents and drugs due to their phospholipid outer layer (Cecchin et al., 2023; Herrmann et al., 2021).

The NIH Small Business Innovation Research (SBIR) and Small Business Technology Transfer (STTR) programs provide seed funding to early‐stage small businesses throughout the nation to bring scientific innovations from the bench to the bedside. Goals of the NIH Small Business programs include stimulating technical innovation, meeting federal research and development needs, increasing private sector commercialization of innovations through federal research and development funding, and fostering and encouraging participation in innovation and entrepreneurship by socially and economically disadvantaged (SDB) persons and women‐owned small businesses (WOSB).

The most common research grant mechanisms used by the NIH Small Business Programs are the R41 and R42 (SBIR), and R43 and R44 (STTR) mechanisms. In Figure 4d, we show the number of NIH exRNA‐related SBIR and STTR awards made over time. While a few exRNA‐related awards were made between 2009 and 2015, we observed a substantial increase in SBIR and STTR awards from 2018 to 2022. This suggests that exRNA‐related technologies are moving beyond basic development and have significant potential for commercialization.

NIH Small Business Program awards are made through both unsolicited and solicited funding opportunities. Unsolicited (or researcher‐initiated ideas) are proposed via the SBIR and STTR Omnibus grant solicitations. However, some SBIR/STTR grant solicitations are focused on specific research areas. For example, in 2017 the National Institute on Drug Abuse (NIDA) released two SBIR/STTR notices of funding opportunities, ‘Extracellular Vesicle Tools, Technologies, and Products for Neuroscience Research’ (RFA‐DA‐17‐008 and RFA‐DA‐17‐009). In 2023, the National Center for Advancing Translational Science (NCATS) released PAR‐23‐267 and PAR‐23‐268 for the ‘Industrialization and Translation of Extracellular Vesicles for use in Regenerative Medicine.’ Solicited awards are indicated in purple (Figure 4) and represent approximately 45% of the total SBIR/STTR awards made in 2017–2022.

**FIGURE 7:**
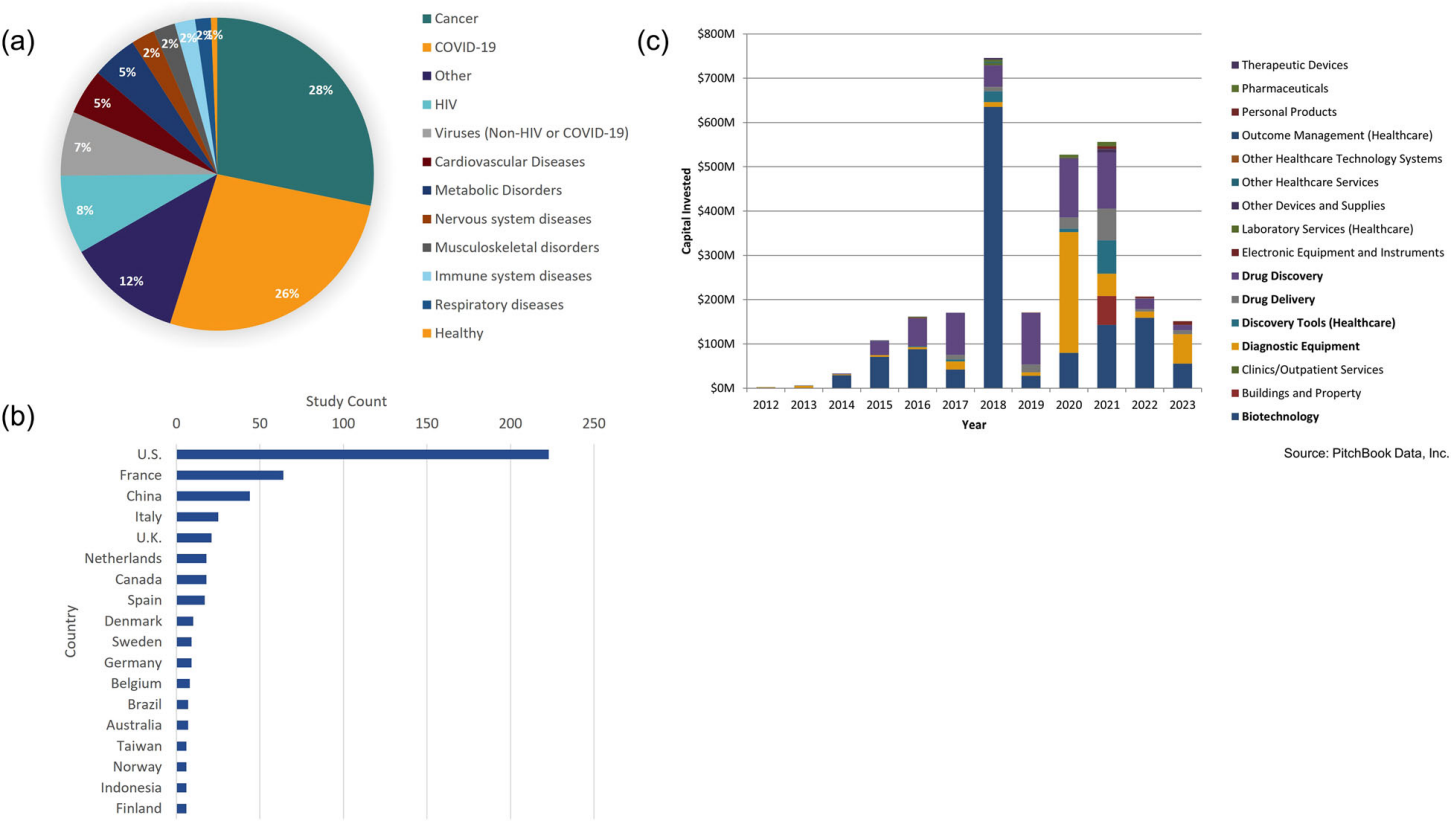
Clinical and commercial application of exRNA and extracellular vesicle‐related research. (a and b) ExRNA and extracellular vesicle‐related clinical trials were identified from clinicaltrials.gov. A total of 1624 clinical trials were identified using a search criteria that included a keyword search of exRNA OR extracellular RNA OR (extracellular vesicle AND RNA) OR RNA communication OR secreted RNA OR RNA trafficking OR circulating RNA OR (Exosome AND RNA). Studies were further limited to statuses of active—not recruiting, available and enrolling by invitation, not yet recruiting, and recruiting. Search date: 9 November 2023. (a) ExRNA and extracellular vesicle‐related clinical trials organized by condition. *n* = 616 (b) ExRNA and extracellular vesicle‐related clinical trials organized by location. Figure depicts countries with >5 ongoing clinical trials, *n* = 504. (c) Commercialization of exRNA and extracellular vesicle technologies. *Source*: PitchBook Data, Inc. A total of 187 companies were identified using a PitchBook search criteria that included a keyword search of ‘exRNA’, ‘extracellular RNA’, ‘extracellular vesicles’ and ‘exosomes’. Companies were assessed for capital invested and then further broken down by primary industry code. Search date: 3 January 2024.

To further understand the commercial market for exRNA and extracellular vesicle‐related technologies, we explored the financial market landscape using the private capital market intelligence platform, PitchBook. According to PitchBook Data, Inc. (as of 3 January 2024) a total of 187 companies are involved in the commercialization of extracellular vesicle, exosome, and exRNA‐related technologies across a number of industries (Figure 7c), representing a total of $2.84B in capital investments in the field. While a majority of the capital invested for the development of extracellular vesicle and exRNA‐related technologies are associated with the biotechnology sector ($1.33B), there has also been significant investment in the areas of drug discovery ($649 M), diagnostic equipment ($456 M), drug delivery ($151 M), and healthcare discovery tools ($113 M) (Figure 7c, indicated by bolding in the figure legend). Capital investments in the development of extracellular vesicle and exRNA‐related diagnostic equipment showed a significant increase in 2020, likely in response to the COVID‐19 pandemic, reaching $272 M in total. This increased investment in extracellular vesicle and exRNA‐related diagnostic technology development has since remained significantly higher compared to pre‐2020 investment levels ($403 M in investments from 2020 to 2023 vs. $53 M between the years 2012 and 2019) (Figure 7c). Taken together, Figure 7c demonstrates significant market investment in the development of extracellular vesicles and exRNA across several industries.

## 3 International efforts

The ERCC was actively involved with international extracellular vesicle efforts throughout its lifecycle. The program invited scientific input from both US‐based and international experts, recruiting External Program Consultants who were experts in their fields to advise on the goals and progress of the Consortium. The External Program Consultants played a vital role in this program by providing feedback on program activities and priorities on a semi‐annual basis. Their feedback helped the program prioritize high impact innovations. Additionally, ERCC PIs had international collaborators associated with their awards, promoting synergy and resource sharing.

The fact that the field has grown significantly in the past ten years is also exemplified by the number of international societies that now exist to promote the development, dissemination, and advancement of knowledge, and to help guide researchers on standards in the field of extracellular vesicles, extracellular particles, and exRNA (Lötvall et al., 2014; Théry et al., 2018; Welsh et al., 2024). These include the International Society for Extracellular Vesicles (ISEV, at isev.org), which at the time of this writing in 2024 is the largest extracellular vesicle society globally and has chapters around the world (available on their Global EV Network page).

The ERCC worked with ISEV and other international extracellular vesicle societies throughout the course of the program. Phase II of the program was informed by data including surveys conducted through both the American Society for Extracellular and Microvesicles (ASEMV) and ISEV. ERCC investigators also partnered with these societies on important efforts such as revising and standardizing extracellular RNA and vesicle communication ontology (Cheung et al., 2016). ISEV and ERCC investigators developed joint educational workshops on topics including exRNA biology, data generation methods, and data analysis, held at ISEV in Toronto, Canada and Kyoto, Japan. ERCC investigators have also been involved with other extracellular vesicle organizations including the Society for Clinical Research and Translation of Extracellular Vesicles Singapore (SOCRATES), the American Society for Intercellular Communication (ASIC), and the American Association of Extracellular Vesicles (AAEV) which include both US‐based and international members. Many ERCC awardees remain actively involved with these organizations, where they will no doubt continue to make key discoveries and develop innovative technologies in the field of extracellular vesicles and exRNA.

The clinical utility of this field is recognized beyond the United States. As shown in Figure 7b above, while the majority of clinical trials are located in the United States, our search also identified clinical trials in several other countries, with the majority occurring in France and China. An additional 33 countries not listed in Figure 7b, have clinical trials listed in clinicaltrials.gov. These countries, including Singapore, Korea, and Switzerland, among others, have less than five ongoing clinical trials in the exRNA space. One of the caveats of this analysis is that the data source was ClinicalTrials.gov, which is maintained by the NIH National Library of Medicine. This source is likely over representative of US studies‐ US laws, regulations, and policies require US‐based sponsors and investigators to submit certain types of clinical trials to ClinicalTrials.gov, while other countries may require deposition into different registries. Therefore, it is likely that there are additional clinical trials in other countries that our search did not identify.

Finally, the exRNA Atlas, an exRNA database developed by the ERCC, has been extensively used by international researchers, indicating that this program has had a global impact on the field. Since the launch of the resource in 2016, researchers from 51 countries outside the US have downloaded 1,075,453 data/metadata files (15,672 GB) of data for analysis as of 31 May 2024. The ERCC monthly Webinar series also featured both ERCC and non‐ERCC researchers from international institutions. Additionally, the ERCC offered an ‘associate membership’ option during the second half of the program allowing both US‐based and international researchers outside the Consortium to join their research efforts.

## 4 Funding emerging topics in exRNA

In alignment with the Common Fund goal of catalyzing discovery and addressing high priority challenges, the exRNA Communication program supported multiple national and international scientific workshops, meetings, and conferences relevant to the program goals. This included Gordon Research Conferences, a Keystone Conference, and support for meetings such as the International Society for Extracellular Vesicles (ISEV) 2015 Annual meeting, and the American Society for Intercellular Communication (ASIC) 2022 Annual meeting. These meetings brought together diverse groups of scientists, including those that are underrepresented in the biomedical sciences, to discuss timely questions in the exRNA and extracellular vesicle field. Furthermore, the 2017 NIH Strategic Workshop on Extracellular RNA Transport helped identify mechanistic and clinical research opportunities for exRNA biology and provided recommendations on high priority areas of research that will advance the exRNA field (Mandel & Metais, 1948). This meeting identified key needs in the field, including an emphasis on rigor and reproducibility of findings. The meeting also focused on addressing technological and scientific roadblocks, such as developing tools and techniques to more reproducibly measure and characterize extracellular vesicles and exRNAs, and using validated model systems to study the biogenesis, transport and function of exRNAs.

## 5 DISCUSSION

The field of exRNA in intercellular communication is rapidly developing. In addition to outcomes from the NIH Common Fund exRNA Communication program, we aim to highlight the evolution of exRNA as a field, as well as emerging topics of interest.

### 5.1 Carriers of exRNA

An important outcome of Phase I of the ERCC was the recognition that there are several distinct carriers of exRNA, both vesicular and non‐vesicular, and that each carrier type contains distinct cargo. This was demonstrated in the most highly cited ERCC exRNA‐related research article (Table 2) from the Coffey group at Vanderbilt (Jeppesen et al., 2019). This publication used two different separation methods to characterize the RNA, DNA, and protein constituents of exosomes and other non‐membrane bound particles. The researchers showed that exRNA, RNA‐binding proteins and other cellular proteins are differentially expressed in exosomes vs non‐vesicle compartments and identified annexin A1 as a specific marker for microvesicles (Jeppesen et al., 2019). By further characterizing the molecular composition of exosome and other exRNA carriers, this publication provided a closer look at the heterogeneity of extracellular vesicles and particles. In 2019, Aleksander Milosavljevic's group took this a step further and used deconvolutional meta‐analysis of ERCC datasets to model exRNA cargo types across five human biofluids (Murillo et al., 2019). Using computational deconvolution, they identified six major exRNA cargo types (Murillo et al., 2019). ExRNA carriers identified in the publication included extracellular vesicles, RNA‐binding proteins (RBPs), and lipoproteins (LPPs), among other unknown nanocarriers. The results of this study indicated that the heterogeneity of exRNA carriers and cargo types exceed the capabilities of the experimental methods to reproducibly isolate and study defined carrier subpopulations and their cargo. The inherent diversity of exRNAs, their nanoscale size and heterogeneity of their carriers was one of the biggest challenges associated with Phase I of the ERCC (Li et al., 2018; van Niel et al., 2018; Zhang et al., 2018). It was also the impetus for Phase II of the ERCC which focused on the separation of exRNA carriers and single extracellular vesicle isolation and analysis (Figure 1). The Phase II goals led to the characterization of several new exRNA carriers. The Coffey group was also the first to identify a distinct extracellular nanoparticle, termed supermeres (Zhang et al., 2021). These non‐vesicular nanocarriers of exRNA contain a number of disease relevant protein cargo and represent another distinct exRNA carrier type. Another recent ERCC publication mapped exRNAs carried by RBPs across a variety of human biofluids (LaPlante et al., 2023). Through computational analysis and experimental validation, 128 exRBPs were identified in at least one biofluid, laying the foundation for a new class of biomarkers. As a biomarker, the ability to detect both RNA and protein within these exRBP complexes provides additional specificity and potentially a higher level of accuracy for monitoring pathological processes and/or early disease detection. However, much remains to be done to not only identify and characterize non‐vesicular exRNA carriers, but to also attribute the biological content of these exRBPs with specific biological action. The future clinical relevance of exRNA and extracellular vesicle‐based biotechnology will require a comprehensive understanding of extracellular vesicle and exRNA carrier heterogeneity, both between and within the different carrier subclasses.

### 5.2 ERCC—A foundation for disease‐specific applications

In 2020, the COVID‐19 pandemic created a public health crisis, and much of the world was mobilized to address the global outbreak. In response to the COVID‐19 pandemic, the NIH launched the Rapid Acceleration of Diagnostics (RADx) initiative to speed innovation in the development, commercialization, and implementation of technologies for COVID‐19 testing (Tromberg et al., 2020). RADx‐radical (RADx‐rad) is one of the four components of the RADx program that focused on accelerating fundamental research development of novel, non‐traditional approaches for COVID‐19 testing and surveillance. While the NIH Common Fund's exRNA Communication program focused specifically on exRNA, the novel technologies developed under the program have the capacity for high‐throughput isolation and characterization of nanoparticles based on biophysical characteristics, including carrier‐specific genomic and proteomic markers. These novel technologies had already established proof‐of‐concept in the isolation and analysis of exRNA carriers through the Common Fund exRNA Communication program. Since exRNA carriers, such as extracellular vesicles and exosomes, resemble many viruses both structurally and functionally, these novel tools and technologies that had already been developed under the program had the potential to be repurposed as SARS‐CoV‐2 diagnostics. NIH released the NOFO, RFA‐OD‐20‐018, to seek new ways to identify the SARS‐CoV‐2 virus using newly developed technologies for single extracellular vesicle, exosome, and exRNA isolation and analysis (Happel et al., 2022). The NOFO aimed to develop highly sensitive, non‐invasive, reliable and reproducible COVID‐19 diagnostic tests. Four awards were made under this RADx‐rad program. This repurposing of exRNA‐related tools and technologies towards the COVID‐19 pandemic demonstrates how NIH Common Fund investment in cross‐cutting areas of science, such as exRNA, can result in the development of innovative tools and technologies for use by the global scientific community.

Besides the NIH Common Fund, a number of NIH ICs have published funding opportunities encouraging exRNA carrier researchers to investigate topics across a number of priority research areas. In the neuroscience realm, these include investigations into the role of central nervous system (CNS) extracellular vesicles in substance use disorders, Parkinson's Disease, HIV infection of the CNS, acute to chronic pain transition, brain development and the blood brain interface (PAR‐20‐147/PAR‐20‐148, PA‐20‐149). The National Institute of Diabetes and Digestive and Kidney Diseases (NIDDK) supported efforts in understanding the role of exRNA carriers in metabolic disorders including obesity and Type 1 diabetes (RFA‐DK‐21‐016). The National Cancer Institute (NCI) supported efforts in the areas of technology development and early cancer detection (PAR‐20‐053, RFA‐CA‐23‐018). Other NIH institutes have promoted research of exRNA carriers in aging or regenerative medicine. In addition to these specific scientific areas, investigators can submit unsolicited, investigator‐initiated proposals to further investigate this topic.

### 5.3 Translation into the clinic

ExRNA and extracellular vesicles have demonstrated clinical applications as both biomarkers and therapeutic agents. Developing the clinical utility of exRNAs was the focus of two programmatic goals of Phase I of the ERCC (Figure 1). The ability of exRNAs to mediate intercellular communication and act as signalling molecules in normal cell homeostasis, or as a consequence of pathological development, makes them excellent candidates for use as biomarkers for the detection and monitoring of disease progression (Happel et al., 2020; Max et al., 2018). This has been demonstrated in multiple ERCC publications (Table 3). In 2016 Freedman et al. demonstrated that diverse exRNAs types [(including microRNA (miRNA), piwi‐interacting RNA, and small nucleolar RNAs] are widely detected in human plasma (Freedman et al., 2016). While miRNA was found to be the most abundant type of exRNA, this report was the first to identify novel extracellular human non‐miRNA small RNAs. It is now known that exRNA is comprised of many different types of RNA and found in all human biofluids (Freedman et al., 2016; Godoy et al., 2018). Combined with the relative stability of exRNAs within the various carriers and the ease of collection, exRNA have enormous potential as clinical biomarkers for the early detection of disease and as predictive biomarkers for therapeutic intervention.

Recently, ERCC researchers and others have shown that the isolation, characterization and selection of extracellular vesicle subpopulations is particularly relevant for the use of exRNA and extracellular vesicle for diagnostic applications. The Das group from Massachusetts General Hospital and the Jovanovic‐Talisman group from the Beckman Research Institute of the City of Hope Comprehensive Cancer Center used quantitative single‐molecule localization microscopy (qSMLM) to demonstrate that cardiac troponin T (cTnT) present in extracellular vesicles has the potential to be a novel biomarker for human cardiovascular diseases (Lennon et al., 2022). The qSMLM technique allowed for the molecular assessment of individual cardiac cTnT‐positive extracellular vesicles, a specific extracellular vesicle subpopulation, and reveals the potential for cTnT to act as a clinical prognostic biomarker. However, further studies are needed to determine the prognostic implications of cTnT‐positive extracellular vesicles. When qSMLM is coupled with affinity isolation, the resulting assay, Single Extracellular VEsicle Nanoscopy (SEVEN) provides a highly sensitive assessment of extracellular vesicle numbers, size, molecular content and shape with their heterogeneities from complex biological samples (Saftics et al., 2023).

Using a different technology, Nguyen et al. explored the presence of programmed death‐ligand 1 (PD‐L1) and programmed cell death‐1 (PD‐1) on extracellular vesicles. To assess and quantify singe extracellular vesicles they developed a novel immunogold biochip technology (termed gold nanoparticle‐based single extracellular vesicular RNA and protein or ^Au^SERP) that has the ability to simultaneously detect proteins and mRNA. Using this ^Au^SERP approach, they showed that PD‐1 and PD‐L1 proteins are present on the surface of extracellular vesicles while they simultaneously detected PD‐1 and PD‐L1 messenger RNA (mRNA) cargo within the extracellular vesicles (Nguyen et al., 2022). PD‐L1 is a protein that is found on some healthy cells but can be overexpressed on some types of cancer cells (Wang et al., 2016). Tumours that express high levels of PD‐L1 may respond well to PD‐1/PD‐L1 directed immunotherapy and tumour PD‐L1 protein levels are used as a predictive biomarker for anti‐PD‐1–directed therapy in many human cancers (Patel & Kurzrock, 2015; Vaddepally et al., 2020; Zdrenka et al., 2024). Using their ^Au^SERP technology, Nguyen et al. found that extracellular vesicle PD‐1/PD‐L1 mRNA biomarkers may be better at predicting patient responses to immunotherapy than the standard Federal Drug Administration (FDA) approved PD‐L1 protein immunohistochemistry assays (Nguyen et al., 2022). These technologies represent a significant accomplishment in the ability to isolate and characterize individual exRNA carriers and provide unique information about the exRNA carriers subtypes. Selectivity is especially relevant for diagnostic applications. These applications can include the ability to sub‐phenotype known diseases and/or the detection of early‐stage diseases. Ultimately, the ability to differentiate disease‐enriched or organ‐enriched extracellular vesicle subpopulations and assess cargo from tissue‐specific extracellular vesicles can improve their capacity to serve as clinical biomarkers.

In addition to their role as novel biomarkers, exRNAs and exRNA carriers (such as extracellular vesicles and exosomes) have significant potential as novel therapeutic targets and as drug delivery vehicles. In a highly cited ERCC publication from 2019 (Table 2), Robert Blelloch's group identified exosomal PD‐L1 as a potential therapeutic target (Poggio et al., 2019). It is known that PD‐L1 on cancer cells engages with PD‐1 on immune cells, contributing to cancer immune escape, and blocking PD‐1/PD‐L1 interactions through immunotherapy can reactivate the anti‐tumour immune response. Blelloch's group demonstrated that PD‐L1 is secreted in tumour‐derived exosomes and that exosomal PD‐L1 promotes tumour growth (Poggio et al., 2019). Further, they showed that blocking exosomal PD‐L1 expression in mice extends survival by promoting anti‐tumour immunity while exogenously expressed exosomal PD‐L1 can rescue immune suppression and promote tumour growth. Overall, they not only uncovered a key role for exosomal PD‐L1 in enabling cancer cells to evade anti‐tumour immunity, but also show that exosomal PD‐L1 represents an unexplored therapeutic target, which could overcome resistance to current immunotherapies that disrupt PD‐L1/PD‐1 interactions and lead to reactivation of the anti‐tumour immune response.

ExRNA carriers have significant promise as drug delivery platforms since exRNAs must be packaged in stable carriers to facilitate delivery of the exRNA to the intended target. Their drug delivery potential is due to the high bioavailability, exceptional biocompatibility and low immunogenicity of extracellular vesicles (Cecchin et al., 2023; Klyachko et al., 2020). Extracellular vesicles can cross biological barriers, can be modified to load molecular drugs, have few known deleterious effects, and can maintain their activity during storage (Du et al., 2023; van de Wakker et al., 2022). Although extracellular vesicles are assembled from a complex mixture of various lipids and membrane proteins as opposed to synthetic nanoparticles traditionally used as drug delivery vehicles, it is the inherent complexity of extracellular vesicles that aid in tissue targeting and ensure minimal non‐specific interactions (Herrmann et al., 2021; Witwer & Wolfram, 2021). These advantages open the door for extracellular vesicles as drug delivery vehicles that can potentially compensate for the drawbacks of synthetic delivery systems. While there is much more to be done to fully realize the potential of extracellular vesicle‐based therapeutics, exRNAs and extracellular vesicles have the capacity to deliver much needed treatments for a variety of human diseases.

The field of exRNA research is expanding beyond fundamental studies towards clinical applications and commercialization. Particularly, extracellular vesicle‐derived exRNAs obtained non‐invasively from liquid biopsies (from biofluids such as blood, saliva and urine) are a potential source for diagnostic and therapeutic applications. Analysis of clinical and commercial applications of exRNA and extracellular vesicle‐related research from clinicaltrials.org identified 1624 clinical trials that are in progress across a variety of disease areas (Figure 7a) (U.S. National Library of Medicine, NA). Many of these studies are focused on cancer (28%), COVID‐19 (26%), cardiovascular diseases (5%), immune system diseases (12%) and neurological disorders (5%). Although cancer and COVID‐19 are the most common disease applications for exRNA and extracellular vesicle studies, Figure 7a shows that exRNA and extracellular vesicle‐related clinical trials are currently being conducted for a wide range of diseases and conditions, from obesity and aging to sepsis. This demonstrates the breadth of diseases that could benefit from the clinical application of exRNA and extracellular vesicles.

Three extracellular vesicle‐based biomarker assays have moved from the lab to the commercial market and received FDA Grants breakthrough device designation. The FDA Breakthrough Devices Program is intended to provide patients and medical care providers with timely access to medical devices that are being developed to treat serious health conditions. The extracellular vesicle biomarkers assays that have received this designation are the Bio‐techne ExoDx Prostate IntelliScore EPI CE, Biological Dynamics's ExoVita Pancreas Assay for early detection, and miR Scientific's Exosome‐based miR Sentinel PCC4 Prostate Cancer test. While these assays are the first to hit the market, 95 of the clinical trials in Figure 7a are evaluating diagnostic tests, suggesting there may be more to come from this young field.

### 5.4 Emerging topics in exRNA and extracellular vesicles

Extracellular vesicles have therapeutic potential as drugs and/or drug delivery vehicles across a wide range of diseases. Extracellular vesicles are already under clinical assessment for therapeutic use, as much of the therapeutic potential of adult stem cell‐based therapies have now been attributed to secreted extracellular vesicles (Gnecchi et al., 2005; Lai et al., 2010; Tsiapalis & O'Driscoll, 2020; van Balkom et al., 2013). This is particularly evident in the area of regenerative medicine, a field that aims to restore the structure and function of damaged tissues via the repair of cells, tissues and organs. Studies have demonstrated that extracellular vesicles derived from stem cells, such as mesenchymal stem cells (MSCs) and induced pluripotent stem cells (iPSCs), have inherent therapeutic potential due to their ability to promote tissue regeneration, suppress inflammation, and regulate the immune system (Gao et al., 2020). Extracellular vesicle‐based therapeutics have demonstrated productive repair/regeneration across a number of different tissues and organs including the regeneration of bone, heart, lung, liver, kidney and skin tissues (Karnas et al., 2023; Wang & Pan, 2023).

Microphysiological systems (MPS), also known as organs‐on‐chips (OoCs) or ‘tissue chips’, are bioengineered microsystems capable of recreating aspects of human organ physiology and function. They are in vitro tools that can provide insights into normal human organ function and disease pathophysiology that have multiple far‐reaching applications in drug discovery and development. Tissue chips have many uses including evaluation of toxicity, safety, efficacy of promising therapeutic compounds, as well as biocompatibility and disease modelling. Within the field of exRNA and extracellular vesicles, tissue chips could be a valuable tool to understand the mechanisms of extracellular vesicle‐mediated signalling and target specificity. For every drug nanocarrier, a comprehensive physicochemical characterization, and its interactions in biological environments must be investigated for the therapeutic context of use. While liposomes have been extensively evaluated for efficacy and biocompatibility both in vitro and in vivo (Pande, 2023), this is not the case for extracellular vesicles. The field of extracellular vesicles can benefit from the three‐dimensional nature, integration of multiple cell types, and biomechanical forces that microphysiological systems provide. Microphysiological systems and extracellular vesicles are currently being combined to investigate both fundamental extracellular vesicle biology and translational application questions (Safarzadeh et al., 2024). The combination of MPS (including multi‐organ tissue systems) and extracellular vesicles could result in a more complete understanding of the mechanisms of extracellular vesicle‐mediated communication and cell and tissue specific targeting that could result in significant advancements for extracellular vesicle‐based therapeutics.

## 6 CONCLUSION

There were many successes as well as many challenges throughout the 10‐year span of the exRNA Communication program. The program brought together researchers across different disciplines and from disparate parts of the United States to form a collaborative and innovative consortium resulting in more than 800 publications that have been cited over 79,000 times. The exRNA Portal, developed by the Data Management and Resource Repository (DMRR), contains exRNA datasets (exRNA Atlas), novel tools (Nanoflow Repository), and resources that are having a transformative impact on the field. A major outcome from the ERCC was the realization that there are many distinct carriers of exRNA both vesicular and non‐vesicular and each carrier type presents distinct cargo. There is now a greater appreciation for the heterogeneity of extracellular vesicles and other nanocarriers, and recognition that exRNA and other cargo are differentially expressed in vesicular versus non‐vesicular carriers. ERCC progress towards the isolation of exRNA carriers contributed to the development of several diagnostic tests to respond to the COVID‐19 pandemic through the RADx initiative, a significant step forward to improve human health and safety. While exRNA and extracellular vesicles have demonstrated potential for clinical applications both in diagnostics and therapeutics, across a number of disease applications, much remains to be done. As the NIH Common Fund exRNA Communication program sunsets, program‐derived publications are still being generated. Thus, the full impact of the program could be better realized and assessed in > 10 years to allow for the dissemination and adoption of the new insights and technologies. This would also allow exRNA and extracellular vesicle‐related applications to be translated into clinically relevant biomarkers and therapeutics. In conclusion, we are confident that the program has contributed fundamental knowledge and resources to the field of exRNA biology, and accelerated progress towards the application of exRNA for future clinical applications.

## AUTHOR CONTRIBUTIONS

**Sara M. Amolegbe**: Data curation (equal); formal analysis (equal); project administration (supporting); writing—original draft (equal); writing—review and editing (equal). **Nicolas C. Johnston**: Data curation (equal); formal analysis (equal); project administration (supporting); writing—original draft (equal); writing—review and editing (equal). **Angela Ambrosi**: Data curation (supporting); project administration (supporting); writing—review and editing (equal). **Aniruddha Ganguly**: Project administration (equal); writing—original draft (equal); writing—review and editing (equal). **T. Kevin Howcroft**: Project administration (lead); writing—review and editing (equal). **Lillian S. Kuo**: Project administration (equal); writing—review and editing (supporting). **Patricia A. Labosky**: Project administration (lead); writing—review and editing (equal). **Dobrila D. Rudnicki**: Project administration (equal); writing—review and editing (equal). **John S. Satterlee**: Project administration (equal); writing—original draft (equal); writing—review and editing (equal). **Danilo A. Tagle**: Project administration (lead); writing—review and editing (equal). **Christine Happel**: Conceptualization (lead); data curation (lead); formal analysis (lead); project administration (equal); writing—original draft (equal); writing—review and editing (lead).

## CONFLICT OF INTEREST STATEMENT

The authors declare no conflicts of interest.

## DISCLAIMER

The views and opinions expressed in this manuscript are those of the authors only and do not necessarily represent the views, official policy or position of the U.S. Department of Health and Human Services or any of its affiliated institutions or agencies.
